# Consolidative use of radiotherapy to block oligoprogression in patients with metastatic melanoma on Systemic Therapy (CURB-Melanoma) - a single arm, phase 2 clinical trial

**DOI:** 10.3389/fonc.2026.1841070

**Published:** 2026-07-06

**Authors:** Oded Icht, Alexander Sun, Philip Wong, Andrew McPartlin, Thiago Pimentel Muniz, Anna Spreafico, Samuel D. Saibil, J. Nicholas Lukens, Sana D Karam, John De Almeida, Marcus O. Butler, C. Jillian Tsai

**Affiliations:** 1 Radiation Medicine Program, Princess Margaret Cancer Centre, University Health Network, Toronto, ON, Canada; 2 Department of Radiation Oncology, University of Toronto, Toronto, ON, Canada; 3 Gray Faculty of Medicine and Health Sciences, Tel-Aviv University, Tel-Aviv, Israel; 4 Department of Medical Oncology and Hematology, Princess Margaret Cancer Centre, University Health Network, Toronto, ON, Canada; 5 University of Pennsylvania, Philadelphia, PA, United States; 6 Department of Radiation Oncology, Washington University School of Medicine, St. Louis, MO, United States; 7 Department of Otolaryngology-Head and Neck Surgery, Princess Margaret Cancer Center, University Health Network, University of Toronto, Toronto, ON, Canada

**Keywords:** immunotherapy, metastatic melanoma, oligometastatic disease, oligoprogression, SBRT

## Abstract

**Purpose/objective:**

Patients with metastatic melanoma on active systemic therapy can develop isolated disease progression while the majority of disease remains controlled (oligoprogression). This phase 2 trial investigates whether stereotactic body radiation therapy (SBRT) or hypofractionated radiation to oligoprogressive sites can improve progression-free survival (PFS) in metastatic melanoma patients receiving first-line systemic therapy.

**Materials/methods:**

This is an open-label, single-arm phase 2 trial. Eligible patients have metastatic melanoma with ≤10 extracranial oligoprogressive sites while on first-line systemic therapy. Oligoprogression is defined using modified RECIST 1.1 or PERCIST criteria. All oligoprogressive sites will receive SBRT per institutional guidelines, typically delivered over 3 fractions. Patients will stay on current systemic therapy. The primary endpoint is PFS defined as the time from enrollment to the date of further disease progression. Secondary endpoints include overall survival, time on current systemic therapy, out-of-field response, tolerance of SBRT, and quality of life (QOL). In addition, serial blood samples are collected for exploratory analyses of circulating tumor DNA (ctDNA), mutant allele fraction, and immune profiling (including CD8+ T-cell subsets). The trial aims to enroll 52 patients over 12 months with additional 24 months of follow-up.

**Discussion:**

This trial will evaluate the impact of SBRT on further disease progression, treatment tolerance, and blood-based immune and molecular correlatives in patients with oligoprogressive metastatic melanoma. Findings may help define the standard of care for managing oligoprogression during first-line systemic therapy.

**Clinical trial registration:**

## Introduction

Oligoprogression is characterized by the progression of cancer at a limited number of sites while majority of the disease remains stable on systemic therapy. Management of oligoprogressive disease may involve either switching systemic therapy or incorporating local treatments such as ablative stereotactic body radiotherapy (SBRT) or hypofractionated radiotherapy, while continuing or modifying the existing systemic regimen.

Prospective data supporting the benefit of SBRT for oligoprogressive cancers are scarce, and no trial has specifically evaluated patients with metastatic melanoma on immunotherapy (1–6). The recently published results of the CURB trial (randomized, phase II) clearly showed a four-fold improvement progression-free survival (PFS) with the addition of SBRT for patients with metastatic non−small cell lung cancer (mNSCLC). The clinical observation was accompanied by a corresponding decrease in mutant allele fraction of circulating tumor DNA (ctDNA) fraction in these patients after SBRT (7). Aside from CURB, another randomized phase 2 trial of SBRT for oligoprogressive cancers of mixed histologies was recently published. The STOP trial randomized patients to either SBRT or continuing standard-of-care (SOC) treatment, but was found negative (6). Thus far, there has been no trial investigating the effect of SBRT in oligoprogressive melanoma.

Melanoma incidence is increasing in Canada and worldwide (8–10). In 2022, an estimated 331,647 persons worldwide were diagnosed with melanoma, and approximately 58,645 persons died of the disease (11).

Although most patients are diagnosed with local disease and are cured with surgical resection, some patients develop or present with metastatic disease. Over the past decade, systemic treatment for metastatic melanoma has changed substantially. For example, the recently published 10-year survival outcomes from the Checkmate-067 trial demonstrated a 10-year mOS of 71.9 months for the combination of nivolumab and ipilimumab, and 37 months of nivolumab monotherapy. The 1- and 2-year PFS were about 50% and 45% respectively (12). For patients who progressed on immunotherapy, treatment options are limited. For patients harboring BRAF V600 mutations, a dual BRAF/MEK inhibitors are considered SOC, with a median PFS (mPFS) of about 10 months (13). Wild-type patients are treated with TIL (tumor infiltrating lymphocytes) therapy, resulting in a mPFS of 7 months (14).

CURB-Melanoma is designed to prospectively assess whether SBRT extends PFS in this population.

## Methods and analysis

### Study design, outcomes and end-points

**Figure 1:**
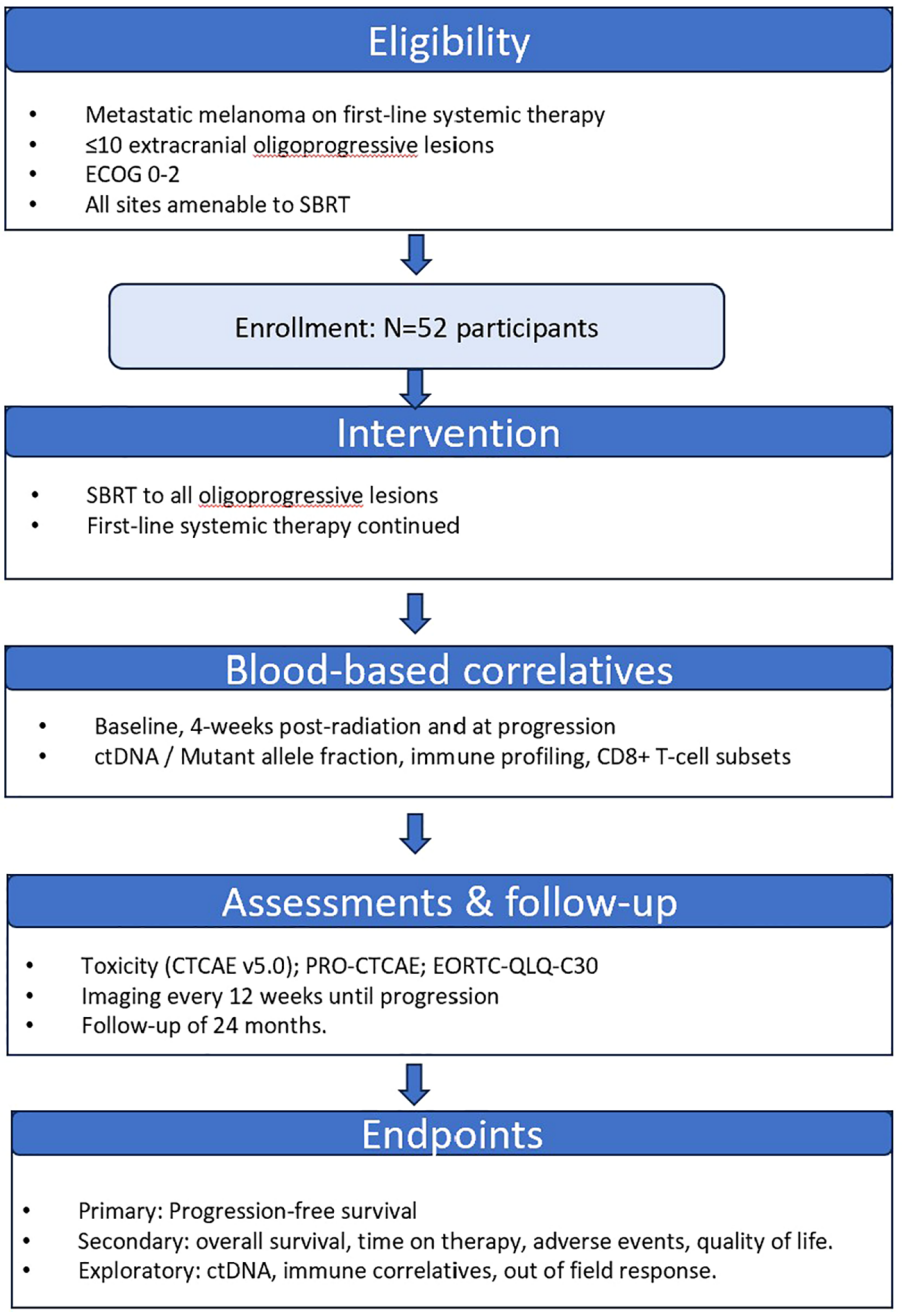
CURB-Melanoma Trial Schema.

CURB-Melanoma is an investigator-initiated, prospective, single-arm phase II clinical trial being conducted at the Princess Margaret Cancer Centre, University Health Network, Toronto, Canada. The trial is enrolling patients with metastatic melanoma who exhibit oligoprogression on first-line therapy (see **Figure 1** for trial schema).

Primary Endpoint:

- Progression-free survival - defined as time from enrollment to further disease progression (assessed by modified RECIST) or death from any cause.

Secondary Endpoints:

- Overall survival - defined as the time from study enrollment to death from any cause.
- Time on current systemic therapy before switching to second line therapy - captures the duration from initiation of the current systemic therapy to the start of second-line treatment.
- Toxicity - treatment-related adverse events will be assessed and graded according to CTCAE v5.0.
- Quality of life - patient-reported outcomes will be evaluated using validated instruments (PRO-CTCAE, EORTC-QLQ-C30).

Exploratory Endpoints:

- Out of field response - defined as a new radiographic response (per RECIST 1.1) in a non-irradiated lesion that was stable at enrollment. This endpoint is hypothesis-generating, as attribution to an abscopal effect is limited by the concurrent influence of ongoing systemic therapy.
- Blood collection: at baseline, 4 weeks post-radiation, and at the time of progression. At each timepoint, two 10 mL Streck Cell-Free DNA Blood Collection Tubes (BCT) will be collected for future analysis of circulating tumor DNA (ctDNA), cytokines, proteomics, metabolomics, and other serum/plasma biomarkers. In addition, two 10 mL Cell Preparation Tubes (CPT) with sodium citrate will be collected for isolation of peripheral blood mononuclear cells (PBMCs) for multiplex analysis of circulating immune cells including, flow cytometry (CyTOF), T cell receptor sequencing (TCR-seq) and single-cell RNA sequencing (scRNA-seq) for a minimal volume of 40 mL.

The primary hypothesis is that adding SBRT or hypofractionated radiotherapy to oligoprogressive lesions while continuing systemic treatment will yield a clinically meaningful extension of PFS. The trial protocol has been approved by the institutional Research Ethics Board, and the study will be monitored by the center’s clinical trials unit for compliance and safety.

### Participant eligibility

Participants must be adults (age ≥18) with an ECOG performance status of 0-2, who have histologically confirmed metastatic melanoma. Patients must be receiving first-line systemic therapy and have demonstrated oligoprogressive disease on restaging evaluations. Oligoprogression is defined in this study as the presence of ≤10 sites of progressive disease (new or enlarging metastases) detected on imaging, while all other disease sites remain stable or responding to therapy. All progressive lesions must be extracranial (patients with only brain progression are excluded) and deemed safely treatable with high-dose radiotherapy.

Inclusion criteria include:

- Metastatic melanoma on first-line systemic therapy: patients may be on anti-PD-1 monotherapy/combination (e.g. nivolumab ± ipilimumab), or on BRAF inhibitors.
- Oligoprogressive disease with ≤10 progressing lesions: progression is defined per modified RECIST 1.1 (≥20% diameter increase) or PERCIST (metabolic progression on PET) criteria for each lesion​. New lesions (≥5 mm) count toward the total of 10​. There is no upper limit on the total number of metastases in the body, as long as at most 10 are progressing.
- Amenable to SBRT: the locations of progression must be such that radiotherapy can be delivered without undue risk (assessed by a radiation oncologist). Lesions previously irradiated can be retreated if safe, and prior palliative radiation is permitted.
- Ability to consent and comply: patients must be willing and able to provide informed consent and comply with study procedures, including completion of quality-of-life questionnaires.

Exclusion criteria include:

- Having >10 progressive lesions (widespread progression).
- Untreated or symptomatic central nervous system disease (patients with leptomeningeal disease are excluded​, as are those with unstable or symptomatic brain metastases).
- Pregnancy, or any serious comorbidity that would contraindicate radiotherapy (such as certain radiosensitive genetic conditions like ataxia-telangiectasia or scleroderma)​.
- Patients in whom prior radiation to a site precludes safe re-irradiation.
- Any circumstance that, in the investigator’s judgment, would interfere with the patient’s ability to participate or with the study results (such as significant psychosocial barriers to follow-up).

### Treatment intervention

Enrolled patients will undergo SBRT or single-fraction hypofractionated radiotherapy to all identified oligoprogressive lesions. The default dose for each oligoprogressive lesion will be 8–10 Gy x 3 fractions, or 8–10 Gy x 1 fraction but other variations are allowed if there are multiple lesions or different dose/fractionations are deemed safer and more feasible per treating physician. For details of planning guidelines please refer to the CURB2 trial protocol [NCT06686771]. Dose and fractionation are individualized per lesion to maximize tumor ablation while maintaining safety (all planned doses are within established normal tissue tolerance limits for SBRT, See **Table 1**).

**Table 1:** Normal structure constraints and compliance criteria (3-fraction regimen).

Serial Tissue	Volume	Per Protocol (Gy)	Endpoint (≥ Grade 3)
Optic Pathway	D0.03cc[Gy]	<=17.4	Neuritis
	D0.2cc[Gy]	<=15.3	
Cochlea	D0.03cc[Gy]	<=20	Hearing loss
Brainstem (not medulla)	D0.03cc[Gy]	<=23.1	Cranial neuropathy
	D0.5cc[Gy]	<=18	
Spinal Cord and medulla	D0.03cc[Gy]	<=22.5	Myelitis
	D0.35cc[Gy]	<=18	
	D1.2cc[Gy]	<=13	
Cauda Equina	D0.03cc[Gy]	<=24	Neuritis
	D5cc[Gy]	<=21.9	
Sacral Plexus	D0.03cc[Gy]	<=24	Neuropathy
	D5cc[Gy]	<=22.5	
Esophagus	D0.03cc[Gy]	<=25.2	Stenosis/fistula
	D5cc[Gy]	<=17.7	
Brachial Plexus	D0.03cc[Gy]	<=26	Neuropathy
	D3cc[Gy]	<=22	
Heart/Pericardium	D0.03cc[Gy]	<=30	Pericarditis
	D15cc[Gy]	<=24	
Great vessels	D0.03cc[Gy]	<=45	Aneurysm
	D10cc[Gy]	<=39	
Trachea and Large Bronchus	D0.03cc[Gy]	<=30	Stenosis/fistula
	D5cc[Gy]	<=25.8	
Bronchus-smaller airways	D0.03cc[Gy]	<=30	Stenosis with atelectasis
	D0.5cc[Gy]	<=25.8	
Rib	D0.03cc[Gy]	<=36.9	Pain or fracture
	D1cc[Gy]	<=28.8	
Skin	D0.03cc[Gy]	<=33	Ulceration
	D10cc[Gy]	<=31	
Stomach	D0.03cc[Gy]	<=24	Ulceration/fistula
	D10cc[Gy]	<=21	
Bile duct	D0.03cc[Gy]	<=36	Stenosis
Duodenum	D0.03cc[Gy]	<=24	Ulceration
	D5cc[Gy]	<=15	
	D10cc[Gy]	<=11.4	
Jejunum/Ileum	D0.03cc[Gy]	<=27	Enteritis/obstruction
	D5cc[Gy]	<=16.2	
Colon*	D0.03cc[Gy]	<=30	Colitis/fistula
	D20cc[Gy]	<=20.4	
Rectum	D0.03cc[Gy]	<=30	Proctitis/fistula
	D20cc[Gy]	<=20.4	
Ureter	D0.03cc[Gy]	<=30	Stenosis
Bladder	D15cc[Gy]	<=15	Cystitis/fistula
Parallel Tissue	Constraint**	Per Protocol	Endpoint (≥ Grade 3)
Lungs-GTV	CV10.5Gy	>=1500cc	Basic Lung Function
	CV11.4Gy	>=1000cc	
	V20Gy	<=15%	Pneumonitis
	V11Gy	<=37%	
Liver	CV17.1GY	>=700cc	Basic Liver Function
Kidneys (Right & Left)	CV15Gy	>=200cc	Basic Renal Function

Treatments are delivered using image-guided radiotherapy on dedicated linear accelerators. SBRT to multiple lesions may be given sequentially or concurrently, at the discretion of the treating radiation oncologist, but the aim is to complete radiation to all sites as soon as feasible, preferably within two weeks of enrollment. Radiation therapy is typically given during the “off week” of systemic therapy to avoid treatment interruption; this timing is logistical rather than biological, and no drug holiday is planned. Thus, patients continue on their systemic treatment beyond progression, with the intent that eliminating the resistant lesions will allow the systemic treatment to remain effective for the other sites. All radiotherapy and medical oncology interventions in this study are standard modalities - SBRT is an established technique, and the drug regimens are the current standard of care for first-line treatment. Supportive care is provided as needed, and any toxicity from radiation or systemic therapy is managed per usual clinical practice.

### Assessments and follow-up

The schedule of study assessments is summarized in **Table 2**. Baseline evaluations: At study entry, baseline tumor assessments are documented using imaging (CT, PET/CT and/or MRI) performed within 6 weeks prior to enrollment​. This imaging serves as the reference for measuring treatment response and progression going forward. Patients also complete baseline patient-reported outcome questionnaires (PRO-CTCAE, EORTC-QLQ-C30). Baseline blood samples are drawn and banked for exploratory biomarker studies.

**Table 2:** Study timeline.

Required investigation	Pre-study (prior to enrolment)	Before 1st fraction of radiation	First follow-up after radiation (4 weeks ± 2 weeks)	Subsequent follow-up (every 12 ± 2 weeks until 104 weeks)
History and physical exam				
ECOG		X	X	X
Weight		X	X	X
Radiology				
CT CAP or PET/CT	Within ≤42 d			X
CT or MRI Brain (optional)	Within ≤42 d			If indicated
Specimen collection				
Blood collection		X	X	At the time of progression
Adverse events				
Survival status			X	X
CTCAE v5.0		X	X	X
Quality of life				
PRO-CTCAE		X	X	X
EORTC-QLQ-C30		X	X	X

Treatment and follow-up visits: During radiation delivery (which spans up to two weeks), patients are seen regularly for treatment management. An acute toxicity evaluation is performed at the end of the radiation treatment course (within a week of completing radiotherapy). Thereafter, follow-up visits are scheduled at approximately 8 weeks post-radiation, and then every 3 months for the first year, or until further disease progression. At each follow-up, a clinical examination and toxicity assessment are conducted. Adverse events are graded according to NCI CTCAE v5.0. In addition, patients complete PRO-CTCAE questionnaires to capture symptoms and side effects from the patient’s perspective, and the EORTC quality-of-life questionnaires are repeated at defined intervals (e.g. 3, 6, 12 months) to assess any changes in global health status and specific symptoms over time.

Radiologic assessments (CT of chest/abdomen/pelvis, with MRI or PET as needed) are performed roughly every 12 weeks following treatment, in alignment with standard melanoma follow-up schedules, to monitor for disease progression. Responses and progression are evaluated by RECIST 1.1 criteria. Any new or growing lesions are documented. If a patient experiences progression outside the originally treated sites (i.e. new sites of disease or growth of previously stable lesions), this will be recorded as an endpoint (end of PFS), and further management (such as a change of systemic therapy) will be at the treating physician’s discretion. The protocol allows for repeat local therapy in some circumstances, but any progression beyond the initial oligoprogressive event generally signals trial completion for that patient (subsequent therapies are recorded for analysis of time to next treatment).

### Statistical analysis

As reviewed in the literature, first-line therapy with immune checkpoint inhibitors results in a mPFS of 11.5 months (12). Our study was designed with the expectation that we would observe a median PFS improvement of about 7 months with addition of SBRT. Assuming an enrolment time of 12 months and follow-up of 24 months, 15% drop out and another 15% of screen failure, a sample size of 52 patients will achieve 85% power at 0.05 significance level to detect a median PFS of 7 months based on a two-sided, single arm log-rank test.

PFS/OS will be estimated using Kaplan-Meier (KM) method and presented graphically. The cumulative incidences of change in systemic therapy and acute/late toxicity will be estimated using the Aalen-Johansen method, where mortality without events of interest will be treated as competing event. Univariate Cox proportional hazard models will also be conducted to examine the association of PFS/OS with patient and tumor characteristics of interest, such as age, sex and the number of oligoprogressive lesions treated, BRAF mutational status, and type of first-line systemic therapy. Similarly, the univariate Fine-Gray competing risk models will be applied to examine the association of time to change in systemic therapy/acute/late toxicity with the patient and tumor characteristics of interests. Multivariable analyses (MVA) will also be conducted to determine the association if applicable. The change in QOL scores at follow ups from baseline (before SBRT) will be analyzed using linear mixed-effect models and the change in scores of >10 points would be deemed a minimal clinically important difference. The other categorical secondary outcomes (e.g. Out of field response, AE) will be summarized using frequency counts and proportion.

An interim analysis will be conducted after the first 25 patients have been enrolled and followed for at least 6 months, to assess safety and early efficacy trends. If ≥30% of patients experience grade 3 or higher toxicities deemed related to SBRT at that time, early termination of the trial will be considered.

## Discussion

The CURB-Melanoma trial evaluates whether SBRT to oligoprogressive lesions, combined with continued systemic therapy, extends PFS in metastatic melanoma. Prospective data in other solid tumors support this strategy: local ablation of limited progression has been shown to prolong duration of first-line therapy.

Oligoprogression, defined as isolated progression in a few lesions amid otherwise controlled disease (15), often reflects clonal resistance confined to select metastatic sites. In those settings, ablating oligoprogressive sites with SBRT prolongs the use of first-line therapy and delays the need for regimen change (16). The principle extends to melanoma: given the durability of responses to immune checkpoint inhibitors (ICIs), strategies that enable continuation of systemic therapy despite focal progression may provide substantial benefit.

The threshold of ≤10 oligoprogressive lesions was selected to maximize the eligible population in a disease where systemic options at progression are limited. Restricting enrollment to smaller thresholds would exclude patients with modest oligoprogression who have few alternatives and in whom continuation of first-line immunotherapy represents a meaningful clinical goal. This threshold is also consistent with contemporary trial design: SABR-COMET-10, a randomized phase III trial evaluating SBRT in patients with 4–10 oligometastatic lesions, is ongoing with results pending (17), and the ARREST phase I trial demonstrated that ablative radiotherapy is safe and feasible even in patients with more than 10 metastatic sites (18).

In metastatic melanoma, ICIs have substantially improved outcomes. Combination therapy with nivolumab and ipilimumab has yielded 10-year OS of 43% (HR 0.53, 95% CI 0.44-0.65) (12). However, resistance develops in many patients, with progression often confined to limited sites. Management typically involves switching therapies (e.g., BRAF/MEK inhibitors in patients with a driver mutation, TIL therapy, or enrollment into a clinical trials). However, maintaining the same systemic treatment while controlling the progressive sites is an appealing option. This approach has been described in several retrospective trials. For example, a multicentre study from France evaluated the role of SBRT in metastatic melanoma patients. The study included 69 patients, of which 29 had oligo-progression. The 1-year PFS was 41%, the mPFS was about 6 months, and the median time to change treatment was 5 months (19).

In another retrospective study, 101 metastatic melanoma patients treated for oligo-progression with SBRT and hyperthermia were followed up for a median of 15.3 months. Although PFS was not reported, the 12- and 24- month local control rates were promising - 93.5% and 88.3% respectively (20). In another study of metastatic melanoma patients treated with immunotherapy, 36 patients were treated for oligo-progression with a loco regional approach. The mPFS was 32 months (21). An observational trial by Chicas-Sett et al. published in 2022 assessed the effect of SBRT and continuation of immune-checkpoint inhibition beyond progression, among patients with metastatic non-small cell lung cancer or melanoma. Of the 50 patients analyzed, 19 had melanoma, and 12 were evaluable. The mPFS for the melanoma sub-group was 21.2 months (22). While the results of these trials are certainly hypothesis generating, they are all retrospective in nature, and are thus subjected to selection bias. They have also incorporated slightly different combinations (for example hyperthermia combined with RT) and have measured different end-points. The CURB-Melanoma is a prospective trial that will assess survival outcomes as well as patient-centered outcomes such as QOL.

Beyond local control for oligo-progressive lesions, SBRT can serve an immunological role, potentially overcoming resistance to first line systemic therapy. Radiation promotes antigen release, increases MHC expression, and can convert the tumor into an “*in situ* vaccine” (23). This effect is most famously illustrated in the abscopal phenomenon, where tumors outside the radiation field regress after local therapy. Though rare, abscopal responses have been reported in melanoma patients receiving radiotherapy with concurrent ICIs (24–26). The hypothesis-generating ‘RadVax’ trial, presented at the 2024 ASTRO Annual Meeting, demonstrated that patients with metastatic melanoma who had progressive disease following ICI treatment experienced both in-field and out-of-field durable responses after receiving SBRT to a single lesion. Immune profiling in this trial revealed dynamic changes in the immune landscape, including an increase in treatment-responsive effector-memory and central-memory CD8+ T cells following SBRT in responding patients - suggesting that local radiation may promote new T-cell priming and help overcome ICI resistance (27). Circulating tumor DNA, immune profiling, and the functional state of CD8+ T cells, including terminally exhausted subsets, are being developed as tools to stratify patients with melanoma and to track response to therapy (28, 29). The serial blood-based correlatives in CURB-Melanoma are intended to capture these measures and to generate hypotheses about which patients derive the greatest benefit from SBRT during first-line systemic therapy.

The rationale for adding SBRT to oligoprogressive disease applies across systemic therapy classes. Independent of the immunological effects of radiation, ablating the limited sites that have acquired resistance allows continuation of an otherwise effective regimen, whether immunotherapy or targeted therapy, and delays the switch to a less effective subsequent line. This local-control rationale is mechanism-agnostic and is the basis for the strategy in BRAF/MEK inhibitor-treated patients. The immunological rationale, including antigen release and abscopal responses, is most directly relevant to patients receiving immune checkpoint inhibitors, but radiation-induced immune priming is not exclusive to this group: BRAF/MEK inhibitors themselves increase antigen presentation and intratumoral T-cell infiltration, and radiation may further augment these effects. With first-line immunotherapy now preferred in most patients, the targeted therapy subgroup in this trial is expected to be small.

Clinical data from other sites of disease also support the synergy between RT and ICIs. For example, in the KEYNOTE-001 secondary analysis, NSCLC patients who had received prior radiation had significantly improved outcomes on pembrolizumab: median PFS increased from 2 to 6.3 months and OS from 5.3 to 10.7 months (30). The CURB-Melanoma trial builds on this evidence. By treating only the progressing lesions while continuing systemic therapy, it offers a way to eliminate resistant clones while leveraging ongoing immune surveillance. This approach avoids premature cessation of a therapy still active in most disease sites. It is analogous to strategies in NSCLC, where ablating progression allows patients to stay on targeted therapy for prolonged periods (16, 31). If similar durability can be achieved in melanoma, SBRT may have a defined role in managing ICI resistance in melanoma.

Toxicity data from trials combining radiation and ICIs have been reassuring. In the recently published phase 2 trial, which investigated SBRT in the oligoprogressive setting, 35% of patients in the SBRT arm received ICIs. Only two grade 3 toxicities were reported, with no grade 4 or 5 events observed (6). Additionally, a phase-1 dose escalation trial assessed the maximum tolerated dose of RT to patients with metastatic melanoma treated with single agent ipilimumab. The patients were treated with 6-8Gy in 2–3 fractions, and found no dose-limiting toxicities related to the radiation/ipilimumab combination (32).

Similarly, low toxicity rates were seen in other tumor types when combining SBRT and ICI treatment (33–35).

This study has several limitations. The most important is the single-arm design. Without a contemporaneous comparator, the contribution of SBRT to any observed prolongation of PFS cannot be isolated from the natural history of the disease or the effect of continued systemic therapy. Patients with oligoprogressive disease likely represent a more favorable prognostic subgroup than the general metastatic melanoma population, and a prolonged PFS in this selected cohort may partly reflect this selection rather than the effect of radiation. The outcomes should therefore be interpreted as hypothesis-generating. A positive efficacy signal would justify a randomized trial with a contemporaneous control arm to establish causal benefit. If the CURB-Melanoma trial demonstrates clinical benefit, it may serve as a foundation for a future phase III randomized trial comparing the current standard approach of systemic therapy modification versus SBRT with continued systemic therapy in patients with oligoprogressive melanoma. Such a trial would address a critical evidence gap and could definitively establish whether metastasis-directed therapy improves not only progression-free but also overall survival in this population.
